# Spatial monitoring of flying insects over a Swedish lake using a continuous-wave lidar system

**DOI:** 10.1098/rsos.221557

**Published:** 2023-05-24

**Authors:** Samuel Jansson, Mikkel Brydegaard, Liang Mei, Tianqi Li, Jim Larsson, Elin Malmqvist, Susanne Åkesson, Sune Svanberg

**Affiliations:** ^1^ Department of Physics, Lund University, SE-221 00 Lund, Sweden; ^2^ Norsk Elektro Optikk AS, Østersjøveien 34, NO-0667 Oslo, Norway; ^3^ Department of Biology, Lund University, Ecology Building, SE-223 62 Lund, Sweden; ^4^ Guangdong Provincial Key Laboratory of Optical Information Materials and Technology and Center for Optical and Electromagnetic Research, South China Academy of Advanced Optoelectronics and; ^5^ National Center for International Research on Green Optoelectronics, South China Normal University, Guangzhou 510006, People's Republic of China

**Keywords:** insects, entomology, ecology, habitat

## Abstract

We have used a continuous-wave bi-static lidar system based on the Scheimpflug principle in measurements on flying insects above, and in the vicinity of, a small lake located in a forested area in Southern Sweden. The system, which operates on triangulation principles, has a high spatial resolution at close distance, followed by a subsequent decline in resolution further from the sensor, related to the compact system design with a separation of transmitter and receiver by only 0.81 m. Our study showed a strong increase in insect abundance especially at dusk, but also at dawn. Insect numbers decreased over water compared to over land, and larger insects were over-represented over water. Further, the average size of the insects increased at night compared to day time.

## 1. Introduction

We present results from non-invasive sampling of optical signals from insects flying over a lake, which normally would be a challenging environment for detailed investigation. The aim of our study is to demonstrate the power of the monitoring technology and to illustrate aspects of the measurement approach in elucidating the behaviour of an insect population. Our laser-based system, which operates in a remote-sensing mode, allows insect monitoring with high spatial and temporal resolution. We could characterize the insects with regard to number and size, and also investigate their preferred habitat.

Insects constitute a very wide variety of species, including strongly beneficial ones providing the pollination necessary for the production of many foodstuffs [1,2]. In tropical regions, insects also constitute vectors for deadly diseases such as malaria, dengue fever and Zika virus [3,4]. Likewise, agricultural pests cause substantial reduction in the yield of crops, and species such as the bark beetle inflict heavy losses in forestry [5–7]. Obviously, monitoring and understanding insects is of utmost importance in order to manage their abundance and influence on society.

Most insects have flying ability during their reproductive stage, which facilitates their monitoring. Traditionally, sweep-nets and traps (sometimes with pheromones) have been used to this end. Radar methods to remotely sample flying insects were demonstrated in the 1970s [8]. Entomological radar systems have subsequently been much refined and are now providing very valuable information on insect migration and dispersal [9,10]. More recently, the optical counterpart to radar, lidar (light detection and ranging) has been developed for insect monitoring. While properties such as wing-beat frequency can be extracted from the data in both cases, the optical regime provides the advantage of adding the spectroscopic dimension, wherein species identification can be improved by analysing reflectance and fluorescence properties, relating to molecular and microstructural information (see, e.g. [11,12]).

Laser monitoring of flying insects was pioneered at Montana State University, where elastic backscattering was used [13,14], and the Lund University group soon extended insect lidar monitoring to fluorescence detection [15], which was also applied to studies of birds [16]. The first studies were performed with conventional time-of-flight lidar systems employing pulsed lasers.

New and strongly extended monitoring possibilities for flying insects were provided when high-power continuous-wave (CW) semiconductor lasers became widely available, and CW lidar systems were developed [17–21]. A bistatic lidar configuration is employed, where target distance is obtained through triangulation. In contrast with earlier bistatic systems, a very short baseline (less than 1 m) is used and allows simple change in measurement direction by turning the whole assembly. Sharp focusing at all distances along the laser beam is achieved by adopting the Scheimpflug principle with a tilted array detector and applying the Hinge rule [17–19]. Several field measurement campaigns have been pursued using such equipment (see, e.g. [20–22]).

The present study regards the employment of a CW Scheimpflug lidar system in monitoring of flying insects over and in the vicinity of the small lake Antragylet in the forested Wildlife Preserve area of Nyteboda in Southern Sweden. We performed measurements during a late June week with warm weather and occasional showers of rain. The recorded insect signals could be segmented with regard to insect size. Special emphasis was put on insect localization with regard to the distance to the nearest shore, revealing the preferred insect flight habitats. The overall flight activity and insect diversity were investigated and compared at the shore and over open water of the lake.

## 2. Experimental arrangements

The experimental set-up and arrangements for the measurements are shown in figure 2. The CW lidar system employed in the experiment was constructed based on the Scheimpflug principle and the Hinge rule [19,23–25]. A CW laser operating at 808 nm with a peak power of 3 W and 50% duty cycle was employed. The laser beam was expanded to a diameter of 120 mm using an amateur astronomic refractor telescope (StarTravel-120, SkyWatcher, Canada) and was in our study transmitted across the lake at a height of about 2 m above the water surface (figure 2*b*). Backscattered light was collected by a 200 mm diameter reflective telescope (Quattro-8C, Skywatcher, Canada), which was mounted on an extruded aluminium bar together with the transmission telescope and a smaller 102 mm diameter refractor telescope for alignment and monitoring of position of the laser beam. The arrangement was supported by a motorized tripod (EQ8, SkyWatcher, Canada), which enabled easy adjustment of the observation direction of the three internally optically aligned telescopes. The system is depicted in figure 2*c*. The laser beam was terminated on a screen of black, matte cardbord with a size of about 0.5 m^3^ (figure 2*a*). This served as a reference point for calibration and was used to secure eye safety. The laser beam diameter at the termination was about 10 cm. We observed that the beam expanded significantly in size at night-time, as caused by dew depositing on the exit lens of the transmitting telescope. An effective remedy was found in wrapping the front part of the telescope with heating tape.

**Figure 1.**
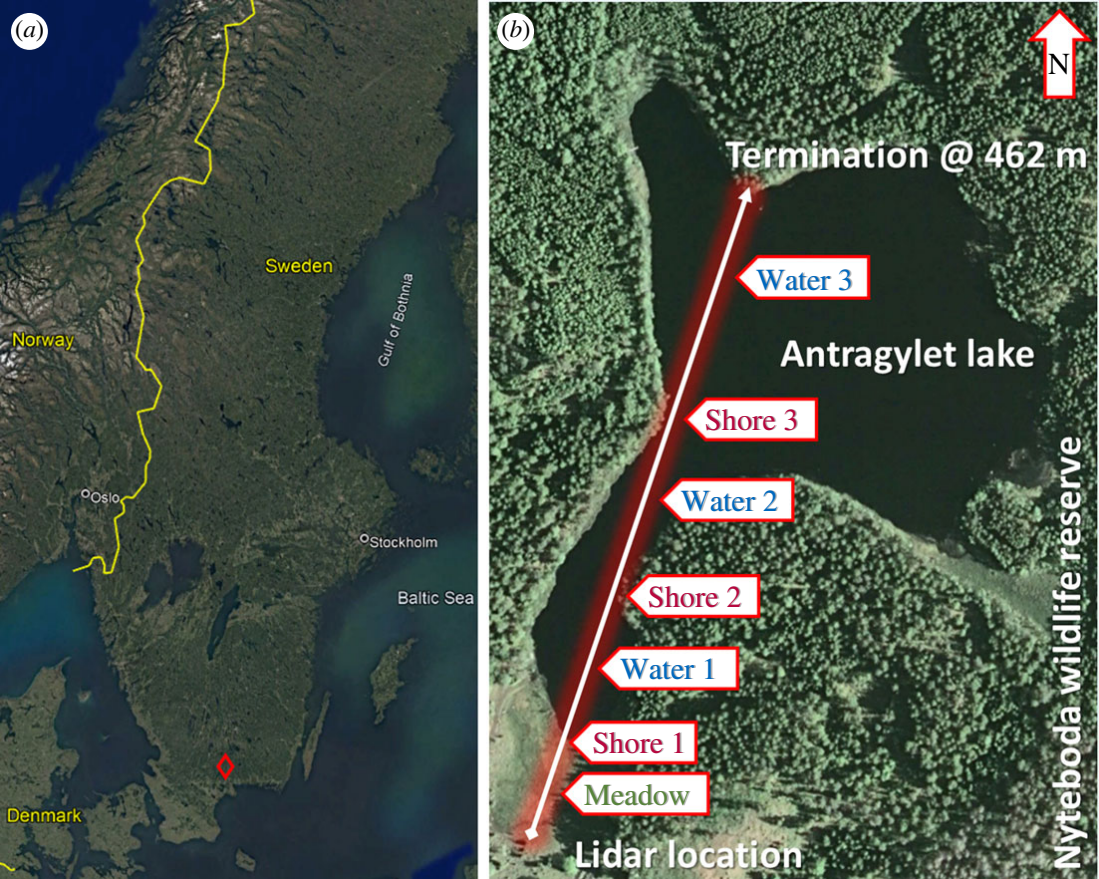
(*a*) The location and satellite map of the Antragylet lake in the Nyteboda forest wildlife preserve in the South of Sweden. (*b*) The location of the lidar system and laser beam termination, as well as topographical features of interest along the laser beam.

The receiving telescope was placed with its optical axis separated from that of the transmission telescope by 81 cm and tilted approximately 1° relative to the laser beam for observation of backscattering along the beam, from a close distance of about 35 m to the termination at a distance of 462 m. Detection was achieved with a USB camera (CCD-S3600, Alphalas, Germany) with a linear array detector (TCD1304DG, Toshiba, Japan) with 3648 pixels of 8 × 200 µm size and a highest read-out frequency of 250 Hz. By switching the laser diode on and off in alternate recordings we could achieve live background light subtraction. Following the optical design rules for a lidar system of this kind (see e.g. [19,24,25]), the detector was tilted to allow sharp imaging of the laser beam simultaneously at close distance as well as far from the lidar. Basically, the simple and well-known rule for camera focusing that the lens has to be moved outwards for close-distance imaging and inwards for far-distance imaging is honoured by the proper detector tilt, achieving exactly the proper diminishing difference in distance between lens plane and detector plane.

## 3. Measurements

We collected lidar data during 27–30 June 2014. The weather was fair on 27 and 30 June, whereas June 28 and June 29 were characterized by frequent rain showers. All periods of rain have been excluded from the dataset, as were periods of (night-time) fog. A typical recording taken directly from the computer screen is shown in figure 3*a*. The range-resolved backscattering intensity is shown from a close distance of 34 m (lower part) to the very strong backscattering echo from the beam termination at a distance of 462 m. Individual echoes of insects traversing the laser beam are seen as recorded on the detetector array during 10 s. Data of this type, as recorded during 4 h, starting at 20.30 and finishing at 00.30 are shown in figure 3*b*. The blips shown in the vertical direction appear as intensity spikes during recording, such as shown in figure 3*a*. We can clearly see the background light level fading out as time proceeds, corresponding to the long summer evenings at northern latitudes. Also, we note that the number of flying insects is very high around the time of sunset around 21.30, and then decreases during the dark night hours. During dusk hours, we also observed regions of pronounced remaining population around 60 and 180 m in range, corresponding to Shore 1 and Shore 2 in figure 1.

**Figure 2.**
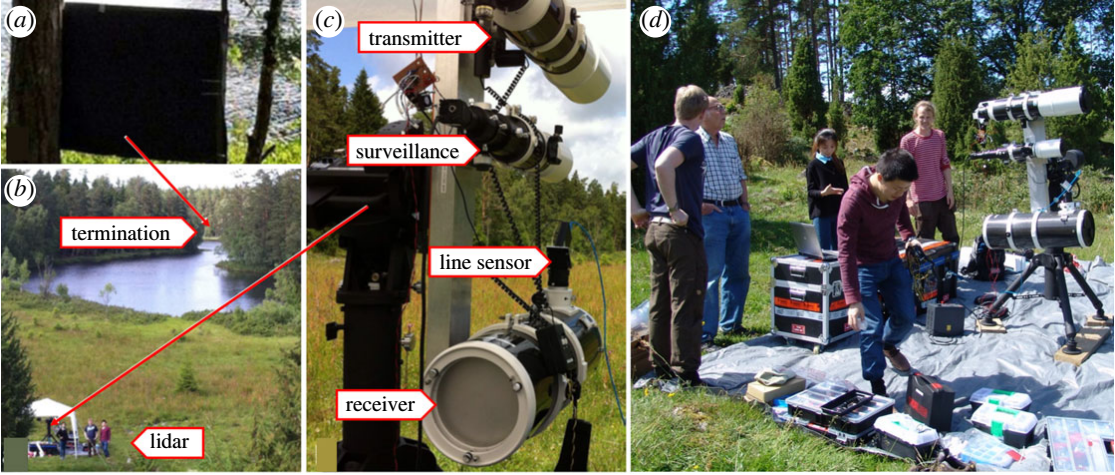
Photographs of the Antragylet experimental site. (*a*) Laser beam black cardboard termination. (*b*) Overview of the lake area with the CW lidar system at the opposite side of the lake. The height of the beam over the lake surface was about 2 m. (*c*) Mounted lidar system. (*d*) The lidar system is seen from the side and referenced in size to some of the authors. The equipment was deployed in the field with a personal car.

**Figure 3.**
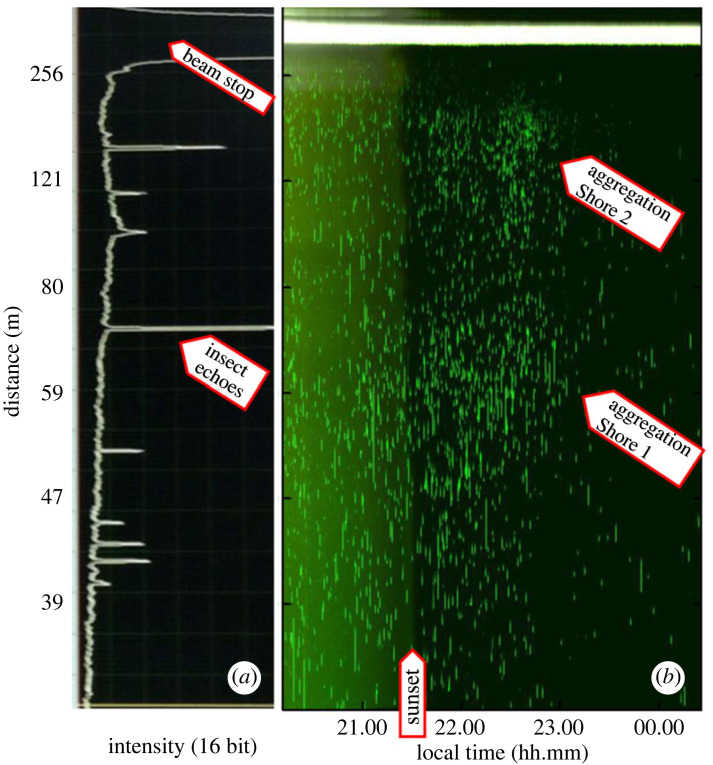
Typical data for flying insects, recorded by the CW lidar system as a function of distance (m). The strong signal at far distance (top) is from the laser beam termination. (*a*) Maximum signal of CCD detector read-outs during a 10 s file. Each peak is the elastic echo from an individual insect crossing the laser beam. (*b*) Data for 4 h of continuous recording. Each green dot is a high-intensity response from the lidar, corresponding to aerofauna entering the laser beam. The elongated echoes are caused by larger insect species or even vertebrates. More point-like echoes are due to smaller insects, such as mosquitos and bark beetles. On the left part of the image (with data up until 21.30), the effect of scattered sunlight is evident in a higher background level that disappears at sunset.

We calculate the apparent size *δ* of each observed aerofauna according to equation (3.1) [25].

**Figure 4.**
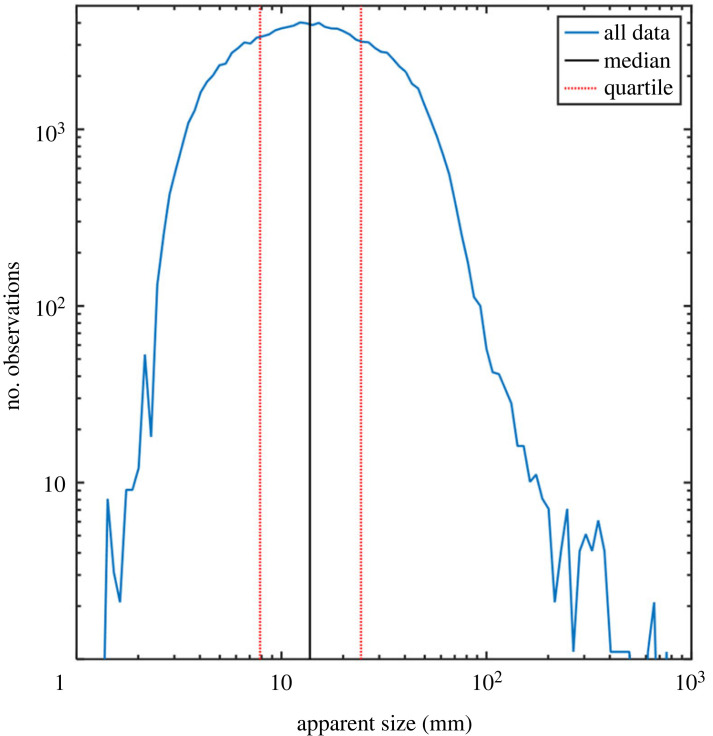
Apparent size distribution of observed aerofauna. The central black line corresponds to the median of the distribution. The IQR of the distribution is indicated. Very large insects are scarce. A few birds and bats passing the laser beam are also observed.

3.1 $$\delta_{\mathrm{obs}}=M\rho n_{\mathrm{pix}}\cos(\varphi_{\det}),$$

in which *M* is the magnification of the telescope at the distance of the observed insect, *ρ* is the pixel pitch of the sensor, *n*_pix_ is the number of pixels by which the target is observed and *φ*_det_ is the tilt angle of the sensor in the Scheimpflug configuration. The apparent size distribution of all observed aerofauna is shown in figure 4.

**Figure 5.**
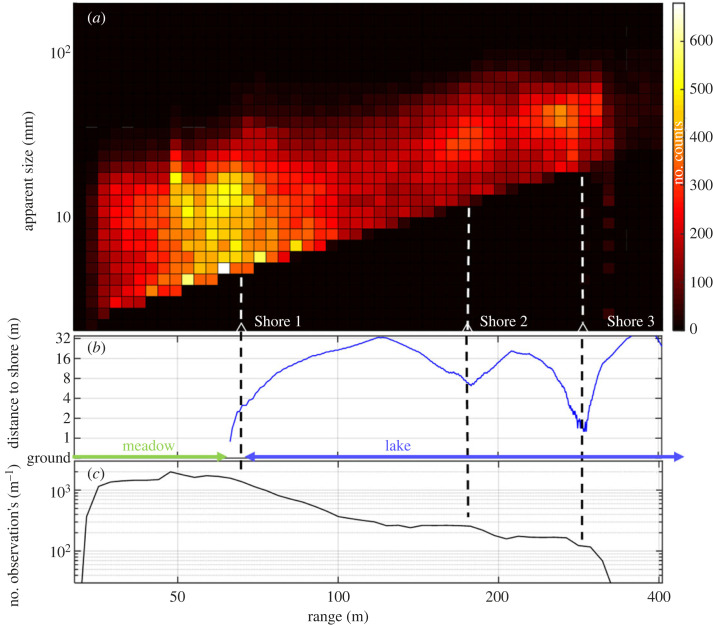
Data from the field experiment. (*a*) shows apparent target size and insect abundance as a function of observation range, while (*b*) maps out the distance from the laser beam to the closest shore. Finally, (*c*) shows the number of observations per metre along the laser beam as a function of range.

**Figure 6.**
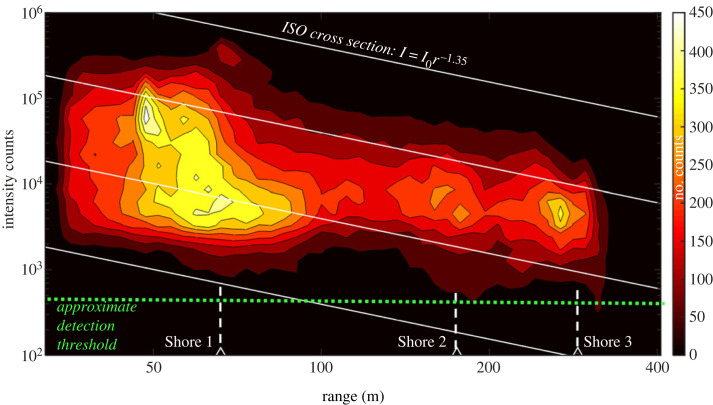
Insect echo strength and number of observed insects as a function of range. System sensitivity fall-off with range, and experimental detection threshold are also indicated.

All data from our study are presented in figure 5 with apparent size plotted versus range in figure 5*a*. Figure 5*b* displays the distance from the laser beam to the nearest shore as evaluated from the Google map data (figure 1*b*). Finally, figure 5*c* at the bottom of the figure shows the number of observations per metre along the laser beam as a function of range. We note that there is an aggregation of observations close to the shores. Actually, the number of counts is observed to fall-off distinctly after the point of closest shore approach. This reflects the combined effect of actual numbers of insects and the fall-off of system sensitivity with range; to be discussed in connection with figure 6. The loss of system sensitivity with range results in smaller objects not being detected at long range.

Figure 6 instead shows the measured signal intensities from flying targets versus range. The system sensitivity fall-off (the form factor) is calculated from the experimentally observed (uniform) air backscattering signal. The air signal is found by a temporal median and is not affected by insects. In our experiment this air signal has a steady increase with an exponent of 0.65 since the beam and pixel foot-print overlap increases for the entire range. Signal strength from scattering Lambertian targets can therefore be expected to attenuate by an exponent of 0.65–2 = −1.35. Such iso cross-section curves are given as straight lines in the log–log plot. The size of insects around Shore 3 can also be observed around Shore 1; however, the smaller insects flying at long range will fall under the system detection threshold.

**Figure 7.**
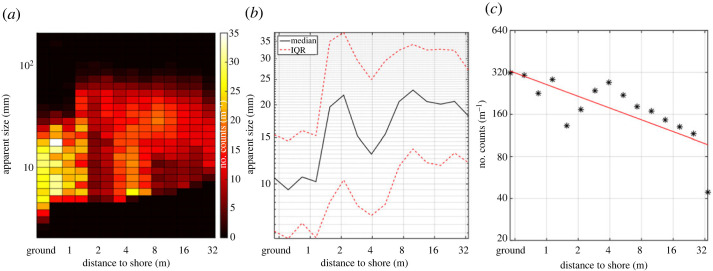
(*a*,*b*) Apparent size and insect count numbers as a function of distance to shore as shown in two different presentations. (*c*) Number of insects observed as a function of distance to shore.

Figure 7 displays data observed in relation to the observation distance from the closest shore. To the left in each diagram, data recorded over ground are given. Figure 7*a* presents how apparent size and the number of counts per metre of the laser beam relate to the distance to the shore. Figure 7*b* is a different illustration of how the apparent size relates to the distance to the shore, with median values and IQR limits (figure 4) for the size indicated. We note that the observed insect sizes increase over the water surface. Finally, figure 7*c* shows how the number of detected insects per metre falls off the further away from the shore the measurements are made. As indicated by the units, all of the subfigures in figure 7 are compensated by the number of metres observed by the lidar transect at different shore distances. A power law was fitted to the abundance versus shore distance (red line in figure 7*c*). We statistically find an $R_{\mathrm{adj}}^{2}$. value of 56% and an exponent of −0.28 (with 95% confidence between −0.42 and −0.14). If the furthest lying point of 32 m shore distance is excluded we obtain $R_{\mathrm{adj}}^{2}=76\text{\%};$ and an exponent of −0.22 (−0.30 to −0.14 with 95% confidence). Therefore, data show a trend significantly different from zero.

Data recorded during different times also show that the average apparent size of observed insects increases in the evening and night as compared to observations made during the daytime. We noted an increase in the most likely size by a factor of 4, from 7 mm to 28 mm, from the afternoon at 16.00 to the night at 23.00. We interpret this observation as a shift to higher abundances of larger night-flying moths, etc. Since observations were interrupted by rain showers and fog, we do not represent any detailed temporal dynamics here.

## 4. Conclusion

We have performed a study of insects flying over and in the vicinity of a small Swedish lake. A remote-sensing optical detection system based on a CW laser in conjunction with an imaging, range-resolving detection system was used for studying the distribution of insects with regard to localization and time. The system allowed insect detection also over water surfaces, which would be difficult to access with traditional techniques. Findings include the following aspects:

1. Insect abundance peaks at dusk and dawn.
2. The number of insects decreases as the distance to the closest shore increases.
3. Larger observations are over-represented over water.
4. As day turns into night, the average size of detected insects increases.

Other entomological lidar studies (see, e.g. [20,21]) confirm that the insect activity is maximal at dusk, and also a secondary activity peak is present before sunrise. Here we could evaluate insect size, and also some vertebrates foraging on the insects were observed. It is our general understanding from this study and previous ones that insects hesitate to fly over open waters, possibly due to the risk of predation by fish, birds or bats [26]. Topographical features such as surface reeds, vicinity to shore or moist meadows increase the abundance of insects significantly. In addition, shade has a large impact on insect abundance, and a lidar transect embedded in a forest structure could display much higher abundance and distinct diurnal patterns.

Our findings clearly relate to observations in other studies. Investigations of movements of different species of insects relative to landscape features are still rare, primarily due to the limitations in methods to study such small animals in the field [18,21,27]. There are several environmental and ecological factors that may affect movement decisions and dispersal capacity in insects [28]. For instance, wind may have a strong influence on movement decisions and concentrations of insects in the landscape, resulting in wind-dispersed movements across open water and land, higher densities near prominent habitat structures, and movements affected by leading-lines [27]. The traversing of open habitats, especially in dry conditions, may cause a higher risk for some insect species, promoting flights in humid weather conditions and under the protection of low light levels and shade. Predation risk from birds and bats may further be increased in open habitats, acting as a selective agent against open habitat movements in insects [21,26]. Habitat fragmentation and increased dispersal costs can have a negative impact on fecundity and lifetime reproductive success in insects [29]. The capacity to disperse has been shown to vary between newly established and old insect populations, with a higher capacity in newly established populations with the result of lower reproduction [29]. Thus, the dispersal capacity and movement adaptations may largely vary between species and ecological situation. Our study shows interesting patterns, where the movements of insects reveal a temporal distribution with highest numbers associated with periods of dusk, but also higher concentrations of smaller insects near the habitat structures and forest edges.

The rapid temporal sampling of this system creates opportunities to study insect responses to transient phenomena, such as wind gusts, turbulence and anthropogenic noise [30–32]. The instrument also enables studies of free-ranging insect responses to sensory stimuli like sound, light and olfactory plumes. Regarding possible influence on the insects by the interogating laser beam, no such effects were observed in our studies. Insects have extended vision towards the ultraviolet region, but lack eye sensitivity in the near-infrared region of laser interrogation [33].

The Scheimpflug lidar approach has proven feasible for field deployment and is an efficient way of observing large numbers of insects and their behaviour with minimal anthropogenic disturbance. We note that if the observed counting rate of 1 per second, as illustrated in figure 3 were maintained through a full 24 h day, this would correspond to about 100 000 observations per day. Clearly, if compared to traditional insect catching using sieves or sweep-nets, the sheer numbers are stunning. Compared to radar techniques in entomology, laser-based remote-sensing systems feature a very narrow transmission lobe, which allows studies even in dense vegetation, where a radar would be fully cluttered by non-relevant echoes. Clearly, an insect lidar can also be operated in a vertically looking mode like most radar systems do. However, a vertical entomological lidar system yields shorter transit times because most insect movements are lateral; also special attention to eye safety issues will then be needed as in other lidar work.

Compared to the present account of the earliest fieldwork on Scheimpflug lidar, subsequent development and studies have improved specificity and contrast between insect classes. The high read-out frequency of novel array detectors allows the capturing of high wing-beat frequencies in the kHz range. Multiple harmonic wing-beat frequencies, which carry additional information on insects, can also be observed. By monitoring polarized light [20,21], information on insect surface structures can be obtained, and by intermittently transmitting laser light of different colours the insect colour/pigmentation is revealed [34].

CW Scheimpflug lidars for different applications are now being used by a number of research groups; see e.g. [23,35–38]. Insect monitoring by further optical techniques is also pursued; see, e.g. [39,40]. More details on optical monitoring of terrestrial and aquatic fauna can be found in a recently published review [12].

The present report sheds new light on what can be expected regarding insect range profiles in relation to forest edges and water bodies. There is an urgent need to understand causes and consequences of biodiversity loss including for insects, and the potential impact of anthropogenic activities on insects numbers (see, e.g. [41]). Therefore, future studies should concentrate efforts on revealing densities, spatial and temporal occurrence of insects in other types of natural and anthopogenic habitats such as grasslands, forests, farms and urban areas.

## Data Availability

Original data are available at Dryad: https://doi.org/10.5061/dryad.9kd51c5n9 [42].
